# An Essay on Diet in Health and Disease

**Published:** 1846-06

**Authors:** John Dawson

**Affiliations:** Jamestown, Ohio


### WESTERN JOURNAL

OF

### MEDICINE AND SURGERY.

JUNE, 1846.

ART. I.—*An Essay on Diet in Health and Disease.* By JOHN DAWSON, M. D., of Jamestown, Ohio.

[CONCLUDED.]

THE DIET IN URINARY CONCRETIONS.

Incident to the performance of their functions, the urinary organs are liable to diseases that are sometimes produced by, and are always more or less under the control of, the food. Among the disorders affecting this apparatus, and the only ones to which we intend to allude, are *urinary concretions.* It is but a few years since Scheele took the first successful step towards the elucidation of this subject. Yet notwithstanding this, his labors in conjunction with those of Wollasten, Pierson, Fourcroy, Vauquelin, Henry, Brande, Prout, Marcet, and Bird, have enriched this field with many new discoveries, and rendered it among the most interesting departments of medical research. The composition of healthy urine has been so accurately determined, that alterations taking place in it from any cause whatever can be very readily and certainly ascertained. Of only some of these, and the conditions of the general system that attend them, is it our present purpose to speak in connexion with the food proper for their correction.

*Lithic or Uric Acid Diathesis.*—In a healthy condition of the organism, it is known that the urine always exhibits acid properties, turning litmus paper red, &c. This, according to Prout, who is high authority on everything connected with diseases of the urinary organs, is not because the fluid contains a free acid, but because the alkaline and earthy bases are not neutralized, but exist in the state of super-salts. The acid properties then depend upon the presence, in the urine, of lithic or uric acid in commbination with ammonia, giving rise to the supersalt called lithate of ammonia. Pure lithic acid is nearly insoluble, and hence, when the ammonia with which it is combined comes in contact with any other acid for which it has a stronger affinity than for the lithic, this latter is precipitated in the form of what is generally known as the lateritious sediment, the particles of which concreting together form gravel, or calculi. To the condition of the system giving rise to this kind of deposit, pathologists attach the name of lithic diathesis. A confirmed diathesis is necessary in order to the production of gravel, which sometimes takes place by accretions in the kidneys, at other times in the bladder.

Nearly always, there is associated with this diathesis a broken down, disordered condition of the digestive functions arising from intemperance, habits of debauche, or gluttony. This state of things disorders all the emunctories more or less, but the kidneys are predominantly affected; and as a consequence elements make their appearance in the urine not proper to this fluid, and which in a normal condition of the system would seek egress in a different direction. Concerning, however, the proximate cause, if it might be so termed, of the urinary deposit under consideration there has been of late much interest manifested, and as a consequence several theories in relation to the subject have been proposed.

Liebig supposes that when from any cause a portion of any tissue becomes unfit for use, or for the performance of its functions, its elements, under the influence of the oxygen conveyed to it in arterial blood, become re-arranged in such manner as to give rise among other compounds to uric acid. More or less of this becomes converted into urea and carbonic acid, the former escaping by the kidneys, the latter from the pulmonary and cutaneous surfaces. Where the circulation is rapid, respiration perfect, and consequently a good amount of oxygen introduced into the system, the change is complete. There is then no excess of uric acid, relatively to the urea, developed. But when these functions are at fault, respiration and circulation being sluggish, the presence of abnormal proportions of uric acid are found in the urine as a deposit. If these views be correct, the presence of uric acid in the kidneys depends upon the imperfect introduction of oxygen into the system. Uric acid, Liebig tells us, disappears in the urine when there is received through the skin and lungs a quantity of oxygen sufficient to oxidize the products of the transformed tissues.

According to Liebig there ought to be an excess of uric acid in the urine of all those animals where there appears to be naturally a slow, imperfect condition of the functions of respiration or circulation; and also in all the diseases where these functions are prominently at fault. So far as some animals are concerned the doctrine holds good. Serpents with a sluggish condition of the respiration and circulation have so much uric acid present in the urine as to render it nearly solid. An opposite condition of the urine is present in carnivorous mammalia, where these functions are more perfect. But how on this hypothesis can the fact be explained, that some birds that have a circulation, respiration, and animal heat superior to man, have so much urate of ammonia in their urine as to render it semi-solid? I allude to those marine birds inhabiting parts of South America, and which cover certain localities completely over with discharges of urate of ammonia, known in commerce as a valuable manure under the name of '*guano.*' Again, according to the same author, we should have the uric acid deposited in all diseases in which the circulation and respiration are below the healthy standard. Becquerel's observations do not confirm such a position. He found in chlorosis the ratio of urea to uric acid equals, and sometimes exceeds, the healthy proportion.—Bird also states that in pulmonary emphysema, in which only an imperfect arterialization of the blood can take place, as is evinced by the blue lips, cold extremities, &c., there is less uric acid found in the urine than in health. Passing to an opposite class of cases, in which, from the more perfect oxidation of the blood caused by the rapid circulation and respiration, there should, if the doctrine under consideration is correct, be a deficiency of uric acid, and an abnormal proportion of urea. Take, for example, phthisis pulmonalis where in the opinion of Liebig the patient is oxidized to death, and what do we find to be the case? Bird assures us that the quantity of uric acid is greater than in health, and the relative proportions of urea less, which is precisely the reverse of what Liebig's theory requires.

Taking it as a well established fact that uric acid exists in the urine in combination with ammonia, Bird supposes that any change occurring which removes the ammonia must necessarily be attended with a deposit of the insoluble acid. By the researches of Prout two acids have been proved to exist in the body, in the free state,—the lactic and the muriatic. They are both found in the stomach, and the lactic is known to be excreted at the cutaneous surface. Now anything interfering with the assimilative functions, or with the action of the emunctories would, as a consequence, derange the excretion of these acids, and compel them, perhaps, to seek an outlet by the kidneys, where, by combining with the ammonia of the urate, they would cause the precipitation of lithic acid in the free state, and thus become a primary cause of gravel.

The opinion of Prout concerning the cause of the deposit under consideration differs but little from that of Bird. Both of these authors, eminent in the literature of diseases of the urinary organs, regard the lithic acid deposit as the result of primary disease, functional and structural, in the assimilative organs attended with a deranged condition of the excretions.

Seguin's experiments on cutaneous transpiration show conclusively that the exhalation from the skin is very much under the influence of the stomach. This author found that the exhalation is always less abundant after taking food; and when the digestive functions are impaired it is sensibly lessened, thus establishing indirectly the opinions of Bird and Prout, that compounds which in health are excreted at the cutaneous surface, are, when the assimilative functions are at fault, either retained in the circulation, or removed by the vicarious office of the kidneys.

With respect to the treatment of the lithic diathesis, all know that it is impossible to dispense with a certain kind of drugs, namely, alkalies. Next, however, in point of importance is the proper selection and regulation of food. Every author of any eminence acknowledges the correctness of this position. Even Liebig, who regards this kind of urinary accretion as the result of derangement of what is sometimes called the secondary destructive assimilation, the re-union of the effete parts of the tissues with the blood, looks upon the proper regulation of diet as an indispensable part of the treatment.

In prescribing food for a person laboring under this diathesis, the objects in view should be, to restore the digestive functions to a healthy condition, and to obviate, as far as possible, the tendency to the formation of free lithic acid. In fulfilling the first indication, the quantity, quality, &c., of the food should be strictly regarded. Where the trouble has ori-

1 *

ginated from excess of food or high living, which according to all observers is frequently the case, the quantity, of course, should be reduced. As Abernethy says, the supplies should be cut off in order that the enemy may vacate the citadel. Too much food at even a single meal gives rise to what is called the *urina cibi*, which deposits uric acid more copiously than either of the kinds called *urina chyli* or *urina sanguinis.* The quantity, therefore, whatever may be the condition of the stomach, must be regulated by the ability which this organ possesses to digest it. Unless completely digested the smallest quantity operates as an exciting cause of the complaint; while on the contrary a quantity that might be considered liberal, may, by invigorating the general powers of the system, operate indirectly as a means of correcting the diathesis.

If it be taken as an established fact that the urine frequently exhibits the physical and chemical properties of the food, there can be left no doubt upon the mind of any, that the quality of the food in the disease before us is entitled to a careful consideration. While all who have any claims to a critical knowledge of urinary deposits, freely admit the truth of the above statement, it is to be regretted that there is not that entire unanimity of opinion in relation to the qualities most available in the treatment, that could be desired. All who favor the opinions of Liebig contend that non-nitrogenized articles, such as those containing large proportions of starch, fat, butter, and sugar, (elements as they suppose of respiration), by combining with the oxygen of the inspired air prevent that peculiar kind of metamorphosis from going on in the tissues that opposes the formation of free uric acid.—These articles, however, are objected to by those who differ with Liebig from other considerations. It is thought by Prout, Bird, and others, that they are contraindicated from the facility with which most of them are first converted into sugar and afterwards into acids. It is with respect to the propriety of food rich in nitrogen that Liebig and Prout are especially at issue. The former says that there are no reasons which support the opinion that nitrogenized food has any influence in the formation of urinary calculi; while the latter assures us that imperfectly assimilated azotised food is often eliminated by the kidneys as urate of ammonia. Bird coincides with Prout on this point, and says, if the ill-digested meal is composed of food rich in nitrogen, as meat or fish, more uric acid, *cœteris paribus*, appears in the urine than if highly carbonized ingredients, as bread, potatoes, &c., had been used. Future experiments must settle this matter in dispute between these illustrious philosophers. In the meantime Prout, from his very accurate knowledge of the subject in general, we should be disposed to take as our guide. To articles of diet or drink containing acids in anything like excess there is pretty general objection. According to all observations they favor the precipitation of the uric acid, and as a consequence are contraindicated. In anything like a good condition of the stomach there is reason to believe that the vegetable acids are digested. But this, in the lithic diathesis, is so seldom the case that they are regarded as a very fruitful source of embarrassment. With respect to the propriety of adopting the most digestible quality of food, there is no diversity of opinion. This is a general rule, without the observance of which, no patient can be benefitted by any plan of treatment.

*Phosphatic Diathesis.*—This state of the system differs widely from that of the lithic diathesis. It is the very opposite, for here the urine is alkaline. Both conditions agree in owing their origin to a faulty condition of the functions of assimilation. On this account it will be unnecessary to do any thing more than merely to advert to the morbid state of the digestive organs, and to the concomitant phenomena that are usually present; for, so far as we know, there are no peculiarities by which the predisposing mischief in the two diatheses can be distinguished.

According to Bird 6·5 grains of phosporic acid leave the system in the urine every 24 hours. The particular state in which this acid exists is a matter of doubt. A considerable portion of it is present in combination with lime, magnesia, and ammonia, forming a double salt, the ammonio-phosphate of magnesia, or triple salt, as it is usually called. These salts, it is supposed, are held in solution in healthy urine by the free acid always present. If, now from any cause the acid falls below the proportion necessary to keep them in a soluble condition, the earthy phosphates are deposited in the urine in the form of a white gravel, occasionally grey; sometimes amorphous, at other times crystalline, and composed generally of phosphoric acid, ammonia and magnesia; sometimes of phosphoric acid and lime. Prout gives an explanation of the pathological chemistry by which the calculous concretion under consideration is formed, that is somewhat different from this. The phosphate of magnesia it is known is contained in healthy urine, and because of its being very soluble is dissolved in that fluid. Under certain circumstances, however, the urea of the urine becomes decomposed in the kidneys and ammonia is extricated, which, combining with the phosphate of magnesia, forms an insoluble compound, the phosphate of ammonia and magnesia. Usually the urine is pale, but when the phosphates are very abundant it becomes turbid; is of low specific gravity; smells differently from healthy urine; is sometimes sufficiently alkaline when voided to turn turmeric paper brown; and becomes after standing awhile putrid, and highly offensive. The more decided and intense the diathesis the more these characters are developed.

To insist on the utility of regimen in this affection is unnecessary. Much of what we have said in relation to the quantity, &c., of the food in the lithic diathesis is applicable here. No medicine, not even nitric or muriatic acid, the main articles relied upon in the treatment, is of any avail unless such measures are adopted as are calculated to give tone and energy to the organs of assimilation. The use of a diet properly regulated is the only foundation upon which any reasonable hope of cure can be predicated. Depressed by the debility resulting from mischief in the blood-making apparatus, the organism is unable to assist the play of affinities by which this accretion takes place, and the only chance of successfully overcoming it, is by removing the cause. By the adoption of a generous diet, and such other hygienic measures as will economise the resources of the system, the object may be accomplished. Articles in this diathesis containing an excess of some of the vegetable acids are by no means contraindicated. On the contrary they may be of some service in overcoming the alkaline state of the urine. From the acknowledged efficacy of opium in rendering alkaline urine acid there is good reason to believe that alliaceous vegetables, if rightly prepared, might prove to be a very valuable kind of diet in this form of urinary disease. Of course, they should be well cooked to deprive them of the acrid volatile oil which they contain.

Concerning the animal aliments best adapted to this affection, we have no diversity of opinion. All agree that the most digestible and nutritious are to be preferred. Mutton, which according to the analysis of Brande contains a greater amount of nutritious matter than the other kinds of flesh in common use, is on this account, where it is in consonance with the feelings of the patient, well calculated to promote the object in view. The flesh of the common chicken, from its nutritive and digestible qualities, is also a very valuable article. For drinks, water made sour with acid wines (claret or hock), or with the aurantaceous fruits, will answer a better purpose than the common beverages, tea and coffee. But although drinks subserve so many purposes in the animal economy, it should nevertheless always be recollected, that they dilute the gastric juice, and if taken too freely impair thus the process of chymification.

*Oxalic Diathesis.*—This is that condition of the system in which there is a tendency in the kidneys to the formation of oxalate of lime, or what is usually called the *mulberry calculus.* The phenomena attending it, and, to a certain extent, indicating it, do not differ materially, so far as the general system is concerned, from what are present in other kinds of calculi. Here also patients are dyspeptic, emaciated, nervous, hypochondriacal, irritable in temper, at times very excitable, deficient in sexual power, and most commonly afflicted with a sense of weight or pain across the loins. The skin, according to Prout, in cases where the diathesis is strongly marked, assumes a dull greenish yellow, or dark olive color. The urine is strikingly characterized by its bright clear appearance, and by being entirely free from deposits, the oxalate being diffused through it in a crystalline form. In addition to the oxalate of lime, the urine, in one-third of all the cases that came under Bird's observation, contained urates in large excess, and in some instances phosphates.

From the fact that most of the symptoms are common also to diabetes mellitus, and from the known facility with which sugar and its chemical allies, starch, gum, and wood fibre, under the influence of oxidising agents, are converted into oxalic acid, Prout came to the conclusion that the oxalate of lime owes its origin to the transmutation of sugar. In his work on physiological and pathological chemistry, Liebig traces the source of oxalic acid not to any changes made upon sugar, but to the oxidation of uric acid. "In France," he tells us, "it is a common occurrence among patients suffering from calculous complaints, that when they go to the country where they take more exercise, the compounds of uric acid, which were deposited in the bladder during their residence in town, are succeeded by oxalates (mulberry calculi), in consequence of the increased supply of oxygen." Bird, in relation to this matter, differs but slightly from Liebig. He concludes that the oxalic acid is the result of a re-arrangement of the elements of urea, in which process there is a portion of oxygen given up.

As all know, nitric acid, from the fact that it is capable of dissolving this kind of concretion, is the drug to be used. And we may judge of the propriety of putting the patient at once upon a suitable diet from the following remarks of

Bird:—that there are "some articles of food at once causing the excretion of this substance (oxalic acid) in great quantities, whilst others have an effect of totally checking it." The food, therefore, should be regulated so as to improve the condition of the digestive organs and prevent, as far as possible, the introduction into the stomach of those articles containing oxalic acid, or which from their chemical composition would be likely to be changed into that substance. The remarks elsewhere made in reference to the proper mode of improving the condition of the stomach are applicable here, and need not be reiterated. But the quantity of the food requires a brief consideration. Aliments containing the vegetable acids are of doubtful propriety. The same may be said of saccharine articles, as well as of everything easily changed into sugar. Those substances containing the oxalic acid, as the young stalks of the rhubarb plant, which are now of such general use in our country, and the *sorrel*, (oxalis acetosella), used so much by the French as a sallad, should be rigidly excluded from the diet. Food well cooked, digestible, and derived in about equal proportions from the animal and vegetable kingdoms, is, all things considered, about as available as any that can be selected. Some attention should be paid to the character of the drinks. Hard water, from the lime which it contains, may exert an unfavorable influence, and on this account, where it is practicable, should be avoided.

### DIET IN TYPHUS FEVER.

Impressed with the importance of so managing the diet in fever of all kinds, as to secure the greatest possible amount of good without producing any injury, have been all the distinguished medical men from the remotest periods of antiquity. Quite early the opinion became prevalent that, in the treatment of this numerous class of diseases, a vast amount of mischief was caused by the improper use of articles designed for nourishment. From frequently witnessing this result, some came to the conclusion, that success depended as much upon what was regarded as judicious regimen as upon medicine.

We will pass briefly in review some of the doctrines held by the ancients on this subject.

Hippocrates "*On the Method of Diet in Acute Disorders*," gives the foundation of all the correct rules which pertain to dietetics in the treatment of fever connected with a high grade of arterial excitement. And so much did he insist upon their strict observance that his plan of treatment, by one, (Asclepiades) has been spoken of "as merely a contemplation on death." Although abstinence was a favorite measure with the Father of medicine in commencing the treatment of what, in his day were called *acute* fevers, he, nevertheless, says that "a diet which is a little too plentiful is much safer than that which is too sparing and thin." Celsus carried his opinions concerning the utility of regimen, perhaps, to an unwarrantable extent. In his judgment food seasonably given, according to Glass's quotation from him, is the best medicine in fever. Valentini also quotes him as saying "Multi magni morbi curantur abstinentia et quiete." Riverius was not behind his day in regard to this matter. "As for the point of nourishment," he remarks, "the diet ought to be thin and sparing in acute fevers. And therein the ancients were so severely diligent as to place the greatest part of the cure in ordering the diet." After this commendation of the plan of the ancients, he goes on to express himself in terms of censure concerning the customs of his own day: "But in our times, at least our country, by the refractoriness of women,, who fear nothing but that the sick persons shall be starved, as all their care in a manner is to cram their children with meat like pudding bags, how empty their brains of wit, or their hearts of grace and wisdom matters not; and the indulgence of physicians, who the best of them smell too strong of the mountebank, it is grown into a fashion in all fevers, the most violent and acute, to allow the sick at all times broths of the flesh of hens, chickens, capons, and mutton."

The eccentric Van Helmont had his own notions about diet. He remarks: "this is the truth of diet which nature doth of her own accord shew and teach and let that thing be one perpetual, that whosoever hath obtained the best remedies of secrets, and he presently restoreth the sick, so also he prescribeth no other diet for sick than for healthy folk. For to the healthy all things are accounted healthy, because the digestive ferments do powerfully draw and restrain all things into their own jurisdiction. And so digestion doth prescribe the rules of diet."

Tissot, a French author of the last century, took very strong grounds in favor of abstinence in all kinds of fevers. "The most observing persons," he says, "constantly remark that when a feverish patient sups what is commonly called some good broth, the fever gathers strength and the patient weakness. The giving of such a soup or broth, though of the freshest and soundest meat, to a man who had a high fever or putrid humors in his stomach, is to do him exactly the same service as if you had given him two or three hours later stale putrid soup." After remarking that it is a very fatal prejudice, under which some labor, of trying to keep up the patient's strength by food, he adds, "the only things which can strengthen sick persons are those which are able to weaken their disease."

In his work "On Diseases of the Army," Pringle makes some very correct remarks in relation to the utility of a nourishing diet in putrid fever. He thinks the putrid diseases, so prevalent anterior to his time, were in a great measure suppressed by the introduction of sugar into general use, which he regards as a very powerful antiseptic. Huxham was also aware of the propriety of a nourishing diet in fever of a low grade of action. He says: "Indeed, as these fevers run very often out to a great length of time, supporting drinks and diet are necessary, without which the patients certainly sink under them."

Cullen notices the prevalence at various periods of putrid fevers, but gives little or no advice in relation to the diet proper in their treatment. Thomas objects to anything in the treatment of malignant and putrid fever, that would be calculated to sustain the system, unless there should be no congestion present.

Fordyce, in his Third Dissertation on what he calls regular continued fever, objects to the use of animal food in all varieties of fever. A very moderate use of farinaceous vegetables is all that seems to him necessary. He says: "If in health food of easy digestion is sufficient to maintain the powers of the body, it is certainly capable of maintaining them in disease, where from the facility of its digestion a greater proportion of it will be converted into chyle than of animal food of much more difficult digestion."

Such are the opinions which, anterior to the time of Armstrong, constituted the literature of dietetics in regard to fever. Hippocrates, Celsus, Riverius, Tissot, Fordyce, Cullen, and Thomas evidently inclined to the side of a spare diet in every variety of fever. Huxham and Pringle advocated the same plan in fever of much arterial excitement; but in the treatment of the epidemic putrid fevers of their day they recommended a nourishing diet from the commencement. So far as we know, they were among the first that succeeded in drawing the attention of the profession to the importance of this useful measure.

At the time that Dr. Armstrong wrote his work on typhus, abstractions from the vital current and everything calculated to waste the energies of the patient, were looked upon by a large portion of the profession as being prejudicial in fevers of a low grade of excitement. The doctrines of Pringle and Huxham were regarded as being correct, and as being confirmed by the results of practice. The ingenious author, however, to whom we have alluded, gave a very unfortunate impulse to the depleting plan of treating typhus and all its allies. Congestion in some form or other, according to him, was the cause of all the debility and prostration incident to the complaint, and as a consequence the obvious remedy, with such pathological opinions, consisted in depletion general and local. The salutary suggestions of Huxham and Pringle were disregarded, and the disease was looked upon as being entirely amenable to antiphlogistic remedies.

Current as this doctrine has been in Europe and America, it is destined to take its place among the things that are obsolete. Observing men every where are becoming satisfied that it is wrong in theory, and wrong in practice. Since the cholera made its appearance, continued fever of every variety is most successfully treated by abstaining as much as possible from free sanguineous evacuations; and by adopting, in everything like typhus, the feeding plan of treatment. This is the testimony of those well qualified to judge, having charge of European and American hospitals; it is also the decision of the skilful part of the profession throughout the country. A question might here be started, whether or not febrile diseases have undergone any material change since the advent of the notorious and fatal pestilence to which we have alluded? As all know, such epidemics do work their impression upon, or modify in some way or other the diseases that succeed them. From the fact, nevertheless, that the correct doctrine relative to adynamic fever obtained, and was successfully tested by practice, anterior to the advent of the cholera, there is good reason for supposing that it is now what it always has been.

We have premised these remarks on the doctrines of the ancients for the purpose more particularly of adverting to the use of food in the treatment of what in our country is called *epidemic typhus fever*. Between the ancients and moderns there is no difference as it regards the propriety of abstinence, or a spare diet in continued fever connected with a high grade of arterial excitement, formerly called acute fever. The teachings of the Father of medicine in regard to such are as valuable as any that have ever been delivered; and are as current at the present time as they were in the days in which the Coan philosopher flourished. Our object is to call the attention of the profession to the sustaining, strengthening plan of treating all febrile disorders in which, from the beginning, there appears to be atony of the principal functions of life.

It may not be out of place here to submit some of the considerations upon which this plan is founded.

In prescribing diet for any malady it is certainly not far wrong to pay attention to some of the features of its pathology, its duration, mode of termination, &c.

In the class of disorders of which we are about to speak, viz: the putrid fever of the ancients; the hospital, jail, or camp fever of Pringle; the nervous fever of Huxham; the typhus of Armstrong; the adynamic gastro-enteritis of Broussais; the typhoid of Louis and Chomel; the follicular enteritis of Andral; the dothinenteritis of Bretonneau, and the epidemic or winter typhus of American writers, there is, among other things, a well marked tendency in the system to debility, marasmus, and disorganization of the tissues. The debility is seen in the general prostration, the weakened action of the heart, the impaired condition of innervation, and in the diminished amount of the secretions. Rapid wasting of the tissues is a very conspicuous phenomenon from the commencement. As is well known, this morbid action extends to every organ and tissue of the system, unless the gelatinous and osseous be exempt, and these certainly are found less altered after an attack of typhus than other parts. By far the greatest change is witnessed in the muscular tissue, and in the adipose deposit. Here from the first onset the phenomena of emaciation are conspicuous, and often continue until the patient is reduced to the condition of a skeleton. More or less disorganization denoting the weakness of the vital force, is present in every case of anything like an aggravated character. Hemorrhage beneath the cuticle, (petechiæ) from the gums, alimentary canal, or the air passages is of common occurrence. Vitality in certain parts of the body at times becomes extinguished, and pieces of flesh mortify, drop out, and thus give rise to ulcers. Besides the alterations in the solids, the blood in disorders of this class is diminished in quantity and depraved in quality. It contains, according to Andral, no spontaneously coagulable matter, is deficient in fibrin and globulin, and presents on inspection a dissolved, putrid appearance.

An enlightened system of dietetics, we think, should take into consideration the *duration* of the malady. In fevers that run their course in a short time the importance of this remark is not so obvious, as in those that are very protracted. As a general rule, the malady before us lasts from seven to twenty-eight days. Prevailing, however, in the form of an epidemic it has a certain cycle of changes through which it will run, and from which it cannot be moved, that in a majority of cases have something like a fixed duration. Very often this exceeds the time at which, in health, without food, starvation would occur. This event we know depends to some extent on the amount of fat in the body, the presence or absence of water, and the degree of motion, voluntary and involuntary, to which the system is subjected. In the complaint under consideration there is reason to believe that it would take place much earlier than in health. The functions of respiration and circulation are increased so much in activity, that there is more oxygen admitted into and transmitted through the system, than in health. This never passes out of the system unchanged; hence the secondary or destructive assimilation, the process of waste, is augmented, as is seen in the rapid emaciation, to furnish the materials with which the oxygen is found combined in the various excretions. Attended, therefore, with such circumstances, there is certainly propriety in carefully noticing the duration of the affection, in order that a kind of food may be provided sufficiently nourishing to sustain the patient until the disease has run its course.

Another view may be taken that seems, in our estimation, to make a timely and judicious administration of nourishing substances proper. We allude to the agency of food in obviating *complications.* All know that inflammations, complicating disease, arise in very opposite conditions of the system. We find them occurring with as much facility in typhus, as in synochus; and it would perhaps be safe to remark, that the more a patient is debilitated the more danger he is in from complications of an inflammatory nature. If this position be correct, things having a tendency to keep the system from sinking into a state of prostration, and the avoidance of whatever is calculated to waste the strength of the patient, merit some attention as the means of averting what, oftener probably than the disease itself, causes a fatal issue.

From such views the sustaining plan of treatment seems most entitled to confidence; but some things, it must be remembered, look plausible in theory which in practice are found to be defective; we do not believe, however, that this is the case in the matter under consideration. Better success will be found to attend the course just mentioned than has attended any other to which the malady has, as yet, been submitted. We shall now proceed to a brief detail of the means proper for carrying this plan into execution.

In making a selection of food it should be recollected that the indications will be best fulfilled by articles digestible, containing a large proportion of nutritious matter, the elements of respiration, and, as far as practicable, those most adequate to exercise an antiseptic influence. As has previously been shown, animal matter contains the greatest proportion of nutritious principles, is least combined with adventitious substances, is as readily assimilated as other kinds of food, and has a composition identical with blood. Possessed of such qualities, it must undoubtedly take the first rank as an available agent in the process of nutrition. In the preparation of the food of course it should be reduced as nearly as possible to the fluid form. Tea, made from the fleshy parts of mutton, beef, or from fowls, answers a very good purpose. Analogous to these, and perhaps equally valuable, are eggs slightly cooked, and milk; the former composed principally of albumen, and the latter rich in casein—elements which have, like the fibrin of flesh, a composition identical with blood.—Besides articles designed for nutrition proper, we want also those which are considered *elements of respiration.* The condition, as we have seen, of the respiratory function is such, that an abnormal proportion of oxygen is introduced into the system, which by combining with the tissues, there is very good reason to believe, is the principal cause of the marasmus. Something, therefore, containing carbon and hydrogen in proportions sufficient to unite with the oxygen of the inspired air, that it may be prevented from acting on the tissues, is what seems, if this view of the matter is correct, to be what is indicated. Vegetables, although not so nourishing as animal food, contains the materials to which we have alluded, in great abundance. Those to be preferred are the amylaceous, found in great abundance in wheaten flour, sago, tapioca, arrow-root, potatoes, &c. The "*cream of ptisan*" of Hippocrates, made from barley by boiling it to the consistence of cream, and straining it, is, we suppose, as valuable a preparation of vegetable food as any of modern origin. In having it prepared this author had his *thin*, *exactly* thin, and *extremely* thin, just as seemed to be required.

With respect to the articles best calculated to exercise a direct antiseptic effect, in order to oppose, as much as possible, the tendency to a diminished cohesion of the solids and fluids, we can say but little. Pringle as we have seen ascribes the decline, in his day, of putrid diseases, such as scurvy, leprosy, dysentery, plague, and pestilential fevers, to the introduction of sugar, beer, and various liquors into general use. It is known, nevertheless, that saccharine articles are very liable to produce flatulence, acidity, and other troublesome symptoms in the alimentary canal. In favor of the utility of beer and several kinds of wine, there is some testimony; but on considering the ambiguous character of any agent in fulfilling the indication before us, it may be concluded that as much can be done to resist the septic tendency by keeping the nutritive process in vigorous action as in any other way.

Stress was laid by the ancients on the *time* during the course of a fever at which the patient should have food. Hippocrates was averse to assigning any particular period as most proper, but insisted that it should be governed by circumstances. Subsequently to his day the subject was agitated by Asclepiades, who fixed upon the fourth day as the most proper period to commence the administration of food. This plan was adopted by Themison, and also by Galen. Among the moderns this question has ceased to be of any interest, as we have consulted none of them by which it is, to any extent, discussed. It is an old adage, nevertheless, that there "is a time for all things," and it may readily be presumed that there is a time at which it would be most proper to commence the administration of food. To the doctrine of Hippocrates we incline, that it should be governed by circumstances. If the patient is very lean it might be advisable to commence giving food at an earlier stage than where an opposite condition obtained. In cases of sudden invasion, characterized by much excitement, abstinence for a few days generally proves beneficial. On the contrary where the disease gradually steals possession of the system, attended with no violence in the symptoms, and from the character of previous cases of a like nature danger of great prostration is apprehended, it would no doubt be well to allow food from the commencement. During the prevalence of an epidemic we can generally learn enough of its pathological habitudes to ascertain the length of time that cases will be likely to run. If this should be great, no time should be lost in getting the patient at once upon a nourishing, digestible diet.

How often should the food be allowed? Frequently this question is put to the practitioner, in his visits to patients laboring under the complaint of which we have been speaking. In attempting to answer it, we might remark that there are two extremes to be avoided. One is to keep from continually teasing the patient with something to eat; the other is in not letting him fast too long. It should be recollected that there are certain physiological laws connected with digestion which it is very important to observe during health. We refer to the necessity of preserving certain intervals between meals, in order to give the stomach time to digest its contents. If, therefore, we desire to save this organ the greatest possible amount of labor, a desideratum in the disease before us, the laws regulating healthy digestion should be carefully regarded. Cramming into the organ every fifteen minutes or half hour food that is even digestible and proper as to quantity and quality, never fails to embarrass the action of a healthy stomach, and of course cannot fail to do the same when the organ is in a state of disease. The other extreme is perhaps equally prejudicial. Disorders arise in the stomach of a healthy person by fasting too long; and other things being equal, there is reason to believe that the stomach after a long fast is not so well qualified to perform its office, as it is after the usual time between meals has elapsed. In addition to this, patients much prostrated need the invigorating influence of diet, in conjunction with other things, to keep them from sinking. From such considerations it is obvious, that, in trying to obtain the greatest possible amount of good from diet, it should be administered, not every few minutes, nor once in twenty-four hours; but after a proper length of time has elapsed to find the stomach in a condition to perform its office. The times at which the patient was accustomed to eat during health, we think, might be selected as being not far from correct. Taken oftener than this, unless where some urgent circumstance makes it necessary, it may fail to do good. Finally, system in regard to this matter is indispensable to success. The vital force, it should not be forgotten, is impaired; and as there is but a small portion of it, in the disease before us, available in the digestive process, it becomes necessary to economise this in many cases with the greatest possible care, as the only means left us of resisting the tendency to fatal prostration.

February, 1846.

